# Enhancement of Abnormal Grain Growth by Surface Quenching Treatment to Eliminate Cu–Cu Bonding Interfaces Using (111)-Oriented Nanotwinned Copper

**DOI:** 10.3390/ma17133245

**Published:** 2024-07-02

**Authors:** Tsan-Feng Lu, Yu-Ting Yen, Yuan-Fu Cheng, Pei-Wen Wang, YewChung Sermon Wu

**Affiliations:** Department of Materials Science and Engineering, National Yang Ming Chiao Tung University, Hsinchu 30010, Taiwan; s0881513.c@nycu.edu.tw (T.-F.L.); yu.en11@nycu.edu.tw (Y.-T.Y.); weddie6231.11@nycu.edu.tw (Y.-F.C.); nycu3116.en11@nycu.edu.tw (P.-W.W.)

**Keywords:** Cu–Cu direct bonding, wrinkled surface, grain growth, quench treatment, strain energy, abnormal grain growth, nanotwinned Cu

## Abstract

Cu–Cu joints have been adopted for ultra-high density of packaging for high-end devices. However, the processing temperature must be kept relatively low, preferably below 300 °C. In this study, a novel surface modification technique, quenching treatment, was applied to achieve Cu-to-Cu direct bonding using (111)-oriented nanotwinned Cu. The quenching treatment enabled grain growth across the Cu–Cu bonding interface at 275 °C. During quenching treatment, strain energy was induced in the Cu film, resulting in a wrinkled surface morphology. To analyze the strain energy, we utilized an electron backscattered diffraction system to obtain crystallographic information and confirmed it using kernel average misorientation analysis.

## 1. Introduction

As Moore’s Law approaches its physical limits for transistor scaling, three-dimensional integrated circuits (3DIC) have emerged as a promising solution to address the need for higher performance, reduced power consumption, and heterogeneous integration in electronic devices. By stacking multiple silicon wafers or dies and connecting them vertically, 3DIC not only allows for a significant increase in the density of transistors, but also shorten interconnect distances, resulting in faster data transfer rates and lower power consumption [1,2,3,4].

To enable 3D integration, two key technologies are essential: through-silicon via (TSV) and wafer bonding. TSV technology entails creating through-holes in silicon wafers and filling them with metal materials to provide conductive pathways [1,2]. Traditional packaging technology uses solder as the joint, which plays a crucial role in stably connecting different chips [5]. However, during the miniaturization process, solder joints encounter several reliability challenges, such as side wall wetting effects, bridge failures, and the formation of fully intermetallic compounds (IMCs). These issues result in increased joint resistance and reduced mechanical properties [6,7,8]. To address these concerns, direct Cu–Cu bonding technique has been proposed [9,10,11]. Cu–Cu joints exhibit excellent electrical properties, thermal conductivity and resistance to electromigration. Moreover, the size of Cu–Cu joints can be reduced to below 10 μm, thereby increasing the density of input/output (I/O) interconnects [11,12,13]. Despite its advantages, Cu–Cu bonding faces several challenges. Cracks can form and propagate along the bonding interfaces, leading to reliability concerns.

The primary mechanism for Cu–Cu bonding is solid diffusion. While elevated temperatures can enhance bond strength by promoting grain growth across the bonding interface [14], they can also cause Si wafers to warp due to the differing thermal expansion coefficients of Si, Cu, and dielectric materials, leading to poor coplanarity [15]. Therefore, inducing grain growth to eliminate bonding interfaces at low temperatures is a widely recognized issue [16,17,18].

Recently, Chu et al. [16] reported that bonding interfaces/interfacial grain boundary (IGB) could be eliminated by using abnormal grain growth (AGG) of highly (111)-oriented nanotwinned Cu. The AGG is driven by kinetic and thermodynamic processes to minimize the grain boundary energy and strain energy [18]. In our previous study, a surface modification technique using epoxy was employed to create a dual-layer microstructure, aiming to eliminate bonding interfaces through AGG of the fine-grained layer [19]. Furthermore, in another experiment, we induced grain growth and eliminated Cu–Cu bonding interfaces through surface quenching treatment [20].

In this study, the previously developed innovative surface modification technique was further applied, introducing strain energy into highly (111)-oriented nanotwinned Cu films, which has significant effects on the AGG of polycrystalline Cu. By providing additional strain energy as the driving force for AGG, we can eliminate the bonding interface in low-temperature Cu–Cu bonding.

## 2. Experimental

### 2.1. Preparation of Nanotwinned Cu Film

For Cu-Cu bonding, highly (111)-oriented nanotwinned Cu (NtCu) films on Si substrates were utilized. The electrodeposition of (111)-oriented NtCu films required (111)-oriented Cu seed layers produced by sputtering. To fabricate the sputtered (111)-oriented Cu film, a 100-nm-thick TiW adhesion layer and a 200-nm-thick Cu film were sequentially sputtered onto a 12-inch Si wafer using an Oerlikon Cluster Line 300. The resulting Cu film exhibited a strong (111) preferred surface orientation. Before electroplating, we cleaned the sample surfaces with acetone and isopropyl alcohol to remove organic contaminants, followed by immersion in citric acid solution to eliminate the surface oxide layer.

The highly (111)-oriented NtCu films were fabricated using a direct current (DC) electroplating process. This method was found by Tao-Chi Liu et al. and has been extensively studied [21]. The electrolyte solution used contains high-purity CuSO_4_ with 0.8 M Cu cations. A high-purity (99.99%) copper sheet served as the cathode. To facilitate the growth of nanotwins, specific surfactants sourced from Chemleaders Inc. (Hsinchu, Taiwan) were added to the electrolyte, along with 40 ppm HCl. Proper stirring of the electrolyte is necessary during electroplating, which was achieved using a magnetic stirring rod (Polytetrafluoroethene, AS ONE, Osaka, Japan) set to 1200 rpm. A DC current density of 120 mA/cm^2^ was applied [22]. After the electrodeposition process, the NtCu films were then planarized using chemical mechanical polishing (CMP). During the CMP process, a favorable slurry (Nouryon, Taipei, Taiwan) was continuously added into a lapper (PM5, Logitech, Scotland, UK). An applied lapping pressure of 1.5 psi and a polishing rate of 0.02 μm/min were set [23].

### 2.2. Sample Pretreatment and Surface Quenching Treatment

After CMP, the samples were diced into 1 × 1 cm^2^ pieces for the bonding process. Two types of NtCu films were used to investigate the effect of the surface quenching treatment on the Cu–Cu bonding interface: NtCu film without surface quenching treatment (referred to as NtCu) and quenched NtCu film (referred to as QNtCu).

During the fabrication of NtCu sample, the sample was subjected to a series of cleaning steps. Initially, ultrasonic cleaning with acetone was performed to remove any contaminants. This was followed by treatment with a citric acid solution to eliminate surface oxides. Subsequently, the sample was rinsed with acetone and deionized (DI) water. The process concluded with a final purging step using N_2_ gas.

**Figure 1:**
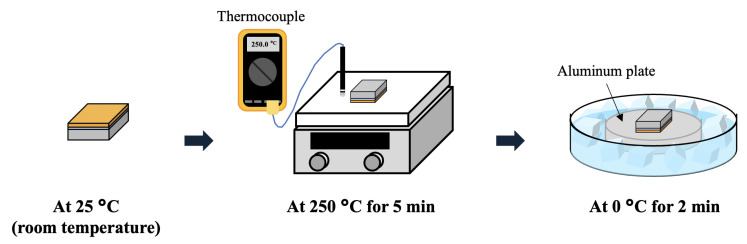
Schematic illustration of the quenching process.

The preparation of QNtCu sample followed the steps used in the fabrication of NtCu sample. First, the NtCu film was heated on a hot plate at 250 °C for five minutes, then rapidly transferred to an aluminum plate at 0 °C for quick cooling over a period of two minutes, as illustrated in Figure 1. Subsequently, to remove surface oxides and contaminants, the sample was immersed in a citric acid solution, rinsed with acetone and DI water, and finally purged with N_2_ gas.

### 2.3. Bonding Process

After completing the sample pretreatment and surface quenching treatment, the samples were placed in a differential thermal expansion fixture made of aluminum and stainless steel for the bonding process. This fixture is the same as the one used in our previous study [19]. To investigate the influence of AGG on bonding interfaces, we bonded samples: Nt/Nt (NtCu-to-NtCu bonding) and QNt/Nt (QNtCu-to-NtCu bonding) at 275 °C for 2, 4 and 6 h, respectively, under ordinary vacuum conditions (1.33 × 10^−1^ Pa). As the bonding temperature increased, the compressive stress on the sample stack also rose due to the differing thermal expansion rates of the fixture’s materials. At 275 °C, the calculated compressive stress was 59.60 MPa. However, determining the actual stress proved challenging due to plastic deformation (creep) experienced by the Cu films at elevated temperatures.

### 2.4. Material Characterizations

Before the bonding process, we measured the root mean square (RMS) surface roughness of the Cu surface using an atomic force microscope (AFM, Bruker Dimension Icon Scanning Probe Microscope, Bruker, Billerica, MA, USA) over an area of 10 × 10 µm^2^. The differences in the surface morphology of the NtCu films, before and after surface quenching treatment, were verified through scanning electron microscopy (SEM) and scanning ion microscopy (SIM) images, using a dual-beam focused ion beam (DB-FIB, Helios NanoLab 650, FEI, Hillsboro, OR, USA). Electron backscattered diffraction (EBSD) using a Nordlys Max3 EBSD detector (Oxford Instruments, Abingdon-on-Thames, UK) was performed to acquire data on the structure, crystallographic orientation, and phase of materials based on Kikuchi patterns. This analysis was conducted using a SEM (JSM-7800F PRIME, Japan Electron Optics Laboratory Co., Ltd., Tokyo, Japan) operated at 20 kV. The orientation imaging microscopy (OIM) EBSD post-processing software (TSL, Inc., Draper, UT, USA) was employed to provide statistical grain size and crystallographic texture data, and it could further analyze local strain and misorientation, such as kernel average misorientation (KAM).

Following the completion of the bonding experiments, DB-FIB, operated at 5 kV, was employed to examine the Cu-to-Cu bonding interface and the grain growth within the bonded samples.

## 3. Results and Discussion

### 3.1. Surface Roughness and Morphology of NtCu Films

**Figure 2:**
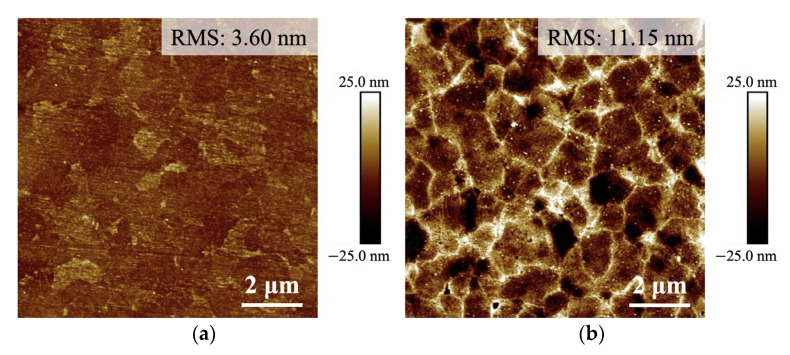
AFM topography images of: (**a**) NtCu and (**b**) QNtCu.

The measured AFM surface topographies of the Cu films are shown in Figure 2. The surface roughness (RMS) of NtCu film and QNtCu film were 3.60 nm and 11.15 nm, respectively. After the surface quenching treatment, the surface roughness of QNtCu film increased, as evidenced by a significant rise in the RMS value.

**Figure 3:**
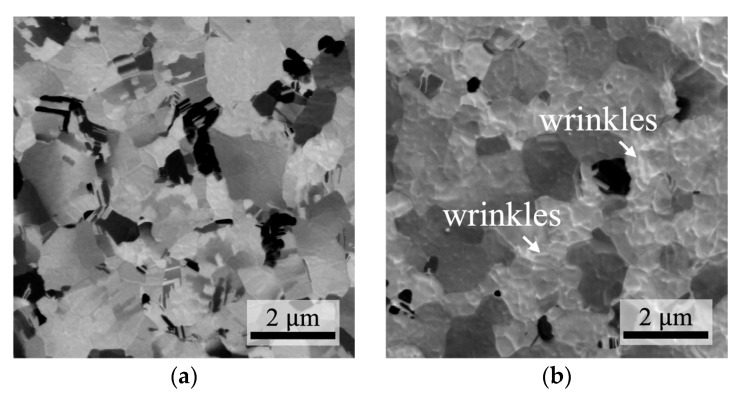

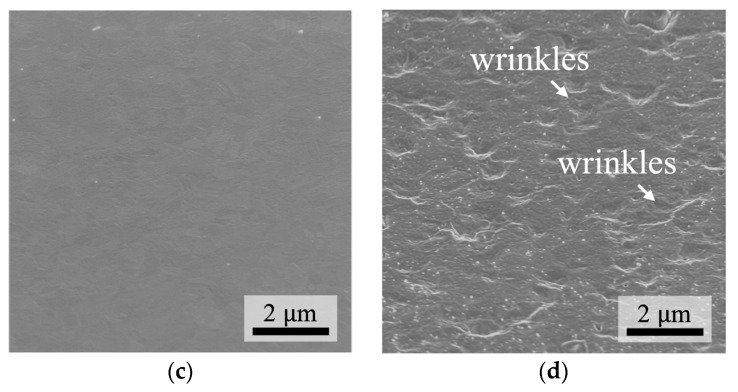
Plane-view SIM images of (**a**) NtCu and (**b**) QNtCu, respectively. SEM images illustrate (**c**) NtCu and (**d**) QNtCu at a 52° tilt. Notably, significant wrinkle morphology is evident in (**d**) following the surface quenching treatment.

FIB analysis was used to investigate the surface morphology. Initially, the NtCu film exhibited a smooth surface morphology, as shown in Figure 3a,c. However, after undergoing the surface quenching treatment, the surface morphology of the QNtCu film became significantly roughened and wrinkled, as shown in Figure 3b,d. The formation of wrinkles on the surfaces of QNtCu films was attributed to the differential thermal expansion between the Si substrate and the Cu film. Rapid contraction of the Cu film during quenching from 250 °C to 0 °C induced strains across its surface. The high strain energy surpassed the activation energy required for wrinkle formation, leading to the observed surface roughness [24,25,26,27]. Contraction stress has the potential to alter local crystal symmetry, causing bond strain and the introduction of crystallographic defects within the Cu film.

This is the reason for the significant increase in surface roughness. It is worth noting that the surface roughness of the QBCu film (quenched electroplated Cu film), with an RMS value of 41.10 nm as reported in previous research [20], is higher than that of the QNtCu film, as shown in Figure 2b. This difference is attributed to the excellent reliability of the NtCu film, which is a result of its high toughness and ductile mechanical properties [28,29,30].

### 3.2. Influence of Strain Energy on Abnormal Grain Growth in NtCu Films

To confirm the presence of inhomogeneous elastic strain energy, we employed kernel average misorientation (KAM) analysis on the surface of the Cu film. KAM, an effective EBSD mode, qualitatively estimates elastic strain based on lattice misorientation. It is calculated as the average misorientation angle between each voxel and its neighboring points in space. For each voxel, misorientation angles exceeding the threshold of 5° are excluded from the average to minimize the influence of high-angle grain boundaries [31,32,33].

**Figure 4:**
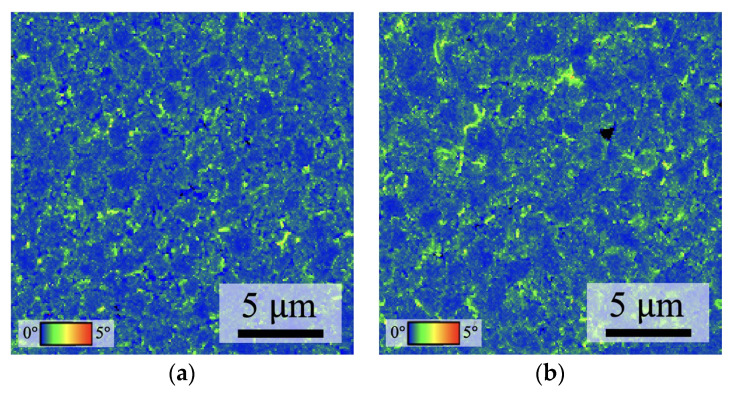
EBSD KAM maps of (**a**) NtCu film and (**b**) QNtCu film. Note: black triangle represents unresolved area.

Figure 4 presents KAM maps of Cu films. In Figure 4a, the majority of the NtCu film appears blue, indicating a low KAM value and dislocation density. Conversely, Figure 4b illustrates that the QNtCu film, after quenching treatment, exhibits a slightly higher KAM value compared to the NtCu film. The average KAM value of the QNtCu film was 0.505, indicating a slight increase compared to the average KAM value of 0.465 for the NtCu film. This increased strain confirms the aforementioned accumulation of strain within the Cu film, which could provide additional driving forces for grain growth during the annealing process, thereby reducing strain energy.

**Figure 5:**
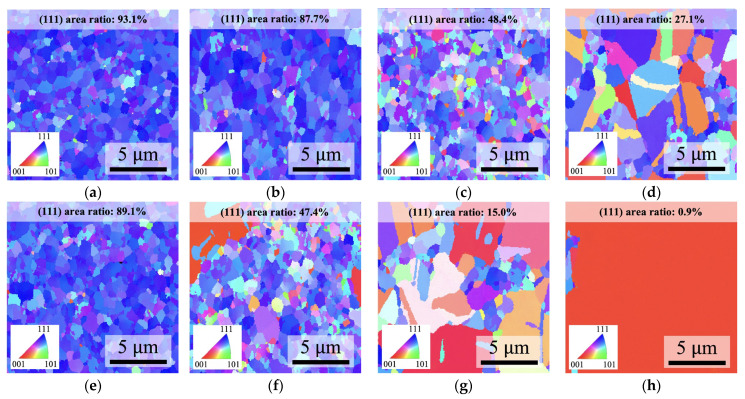
Plane-view EBSD OIM images of NtCu and QNtCu films after annealing at 300 °C for various hours. (**a**–**d**) NtCu: (**a**) As deposited, (**b**) Annealed for 1 h, (**c**) Annealed for 2 h, and (**d**) Annealed for 4 h. (**e**–**h**) QNtCu: (**e**) As deposited, (**f**) Annealed for 1 h, (**g**) Annealed for 2 h, and (**h**) Annealed for 4 h. The (111) area ratio is individually marked in each figure.

To confirm the previously mentioned additional driving forces for grain growth during the annealing process, we employed EBSD OIM images to examine the changes in the microstructures of the Cu films over different annealing times. The NtCu and QNtCu films were annealed at 300 °C for 1 h, 2 h, and 4 h. The colors in Figure 5 represent surface crystallographic orientation. Figure 5a,e show plane-view EBSD OIM images of the initial states of the NtCu and QNtCu films, respectively. Both films exhibit a high preference for the (111) orientation, represented by the blue regions. The (111) area ratio is measured at 93.1% for the NtCu film and 89.1% for the QNtCu film.

When annealed at 300 °C for 1 h, as shown in Figure 5b,f, the EBSD OIM images indicate a decrease in the (111) area ratio to 47.4% for the QNtCu films and 87.7% for the NtCu film, respectively. AGG was observed on the surface of the QNtCu film, while the NtCu film showed no significant variation. Next, we examined the microstructure of the Cu films annealed at 300 °C for 2 and 4 h. It can be observed that the (111) area ratio of both the QNtCu film and the NtCu film decreased with increasing annealing time, as shown in Figure 5c,d,g,h. Specifically, the decrease in the (111) area ratio of the QNtCu film was greater than that of the NtCu film. It is noteworthy that we can observe the majority of (111) grains transforming into (200) grains through AGG in the QNtCu film after annealing at 300 °C for 4 h. During the annealing of the NtCu films, the main reason for the transformation of (111) grains into (200) grains is the increase in stress/strain, leading to the growth of (200) grains to minimize strain energy, as (200) grains possess lower strain energy compared to (111) grains [34,35].

The above results indicate that annealing of the QNtCu film can promote AGG during the annealing process, attributed to additional elastic strain stored in the film. This would facilitate low-temperature bonding and eliminate bonding interfaces (as will be confirmed in the subsequent sections).

### 3.3. Influence of Strain Energy on Abnormal Grain Growth at the Bonding Interface

**Figure 6:**
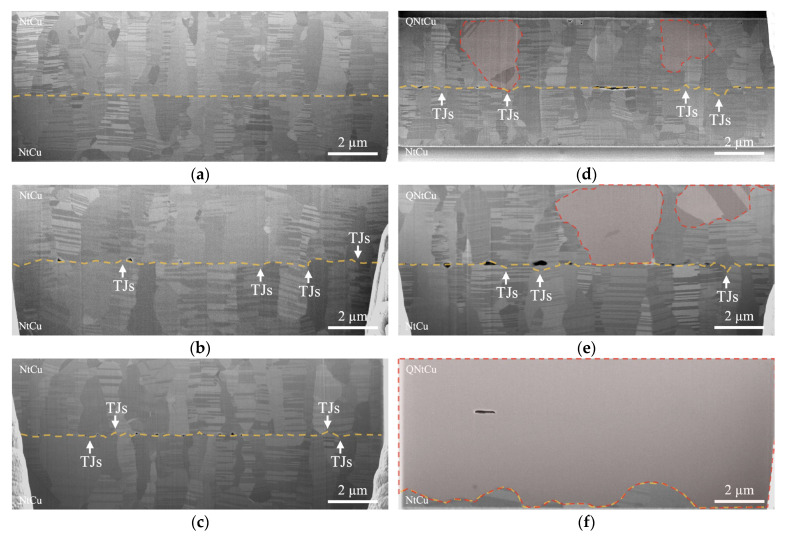
Cross-sectional SEM images: Nt/Nt bonded at 275 °C for (**a**) 2 h, (**b**) 4 h, (**c**) 6 h; QNt/Nt bonded at 275 °C for (**d**) 2 h, (**e**) 4 h, and (**f**) 6 h. Note: The orange dashed lines represent the bonding interfaces/IGB in (**a**–**f**), while the white arrows point to the sites of triple junctions (TJs), in (**b**–**e**). Additionally, the red dashed areas represent AGG in the Cu film in (**d**–**f**).

Grain growth extending across the bonding interface plays a crucial role in determining the mechanical and electrical properties of the Cu joints. Figure 6 presents the microstructures of the bonded interfaces for samples fabricated at 275 °C for 2, 4 and 6 h, with and without surface quenching treatment. Notably, voids were observed at the bonding interfaces, especially in the QNt/Nt sample, as depicted in Figure 6d–f. These voids are believed to be a result of the pronounced roughness of the QNtCu surface prior to bonding (Further evolution of the void has been discussed in previous studies [36,37]).

For the Cu–Cu bonding samples fabricated at 275 °C for 2 h, the QNt/Nt sample exhibited a bonding interface with a zigzag shape caused by bonding interfaces/IGB migration. Additionally, AGG was observed on one side of the QNtCu film, as indicated by the red dashed areas in Figure 6d. However, a distinct and flatter bonding interface was observed in the Nt/Nt sample, which retained a microstructure almost identical to that of the as-deposited NtCu films, as shown in Figure 6a.

As the bonding time increased to 4 h, expanded ranges of AGG occurred in the QNt/Nt sample, with columnar grains gradually consumed from the top of the QNtCu film to the interface, as illustrated in Figure 6e. This result is similar to the EBSD analysis mentioned above for annealing at 300 °C for 1 h, as shown in Figure 5b,f. The (111) area ratio at the bonding interface on the QNtCu side was reduced, while the bonding interface on the NtCu side maintained a high (111) area ratio. However, it should be noted that the EBSD analysis was performed without pressure, which may lead to more significant AGG. The primary reasons for this difference need to be further investigated in future studies. However, no AGG was still observed in the Nt/Nt sample, as shown in Figure 6b.

When the bonding time increased to 6 h, significant AGG occurred in the QNt/Nt sample, leading to the elimination of the bonding interface. Most columnar grains transformed into a single, very large grain, with only the bottom of the columnar structures remaining, as shown in Figure 6f. This result directly confirmed that AGG initiated from QNtCu film and grew downward. However, the columnar structures remained intact, and no AGG was observed in the Nt/Nt sample, as shown in Figure 6c. This is because nanotwins can stabilize the grain boundary structures, resulting in grain growth occurring at a higher temperature than regular Cu. Therefore, introducing additional strain energy through surface quenching treatment facilitates AGG in NtCu film.

From the above results, we observed that AGG initiated from the QNtCu films, attributed to the strain energy introduced by the surface quenching treatment, which facilitated AGG in the QNtCu films and released the stored energy during the bonding process.

Additionally, the zigzag shape phenomenon tends to initiate at triple junctions (TJs) [38,39], forming wedges among three grains, as shown in Figure 6b–e. Before migration, the TJ is the point where a straight IGB intersects with the normal grain boundary of the base material. The transition occurs due to the high energy associated with the T” type grain boundary junctions at the Cu–Cu bonding interface. To decrease this energy, the system rearranges the triple junctions to create a uniform distribution of grain boundary angles. Consequently, the triple junction will migrate to lower the grain boundary energy, leading to the formation of a stable wedge among the three grains.

## 4. Conclusions

In the study, we focus on the driving force of AGG in NtCu films. We found the increase in elastic strain energy after quenching treatment by kernel average misorientation analysis. Furthermore, annealing experiments on the QNtCu films (without pressure) confirmed that surface modification promotes abnormal grain growth. Successful elimination of bonding interfaces and formation of large grains at 275 °C were achieved in Cu–Cu bonding. Through this novel approach, we achieve low-temperature Cu–Cu bonding while effectively eliminating the original bonding interface, thereby enhancing bonding strength and reliability. The electrical conductivity and mechanical strength of the Cu joints, which may be improved by our surface quenching modification process (QNtCu), represent an advancement for 3D IC manufacturing technology, offering potential for future developments.

## Data Availability

The data supporting the findings of this study are available from the corresponding author upon reasonable request.
